# Fixed-target serial crystallography at the Structural Biology Center

**DOI:** 10.1107/S1600577522007895

**Published:** 2022-08-17

**Authors:** Darren A. Sherrell, Alex Lavens, Mateusz Wilamowski, Youngchang Kim, Ryan Chard, Krzysztof Lazarski, Gerold Rosenbaum, Rafael Vescovi, Jessica L. Johnson, Chase Akins, Changsoo Chang, Karolina Michalska, Gyorgy Babnigg, Ian Foster, Andrzej Joachimiak

**Affiliations:** a Structural Biology Center, X-ray Science Division, Argonne National Laboratory, Lemont, IL 60439, USA; b Center for Structural Genomics of Infectious Diseases, Consortium for Advanced Science and Engineering, University of Chicago, Chicago, IL 60667, USA; c Data Science and Learning Division, Argonne National Laboratory, Lemont, IL 60439, USA; d Biosciences Division, Argonne National Laboratory, Lemont, IL 60439, USA; e Department of Biochemistry and Molecular Biology, University of Chicago, Chicago, IL 60367, USA; RIKEN SPring-8 Center, Japan

**Keywords:** fixed-target serial synchrotron crystallography, X-ray free-electron lasers, structural biology

## Abstract

Serial synchrotron crystallography enables the study of protein structures under physiological temperature and reduced radiation damage by collection of data from thousands of crystals. The Structural Biology Center at Sector 19 of the Advanced Photon Source has implemented a fixed-target approach with a new 3D-printed mesh-holder optimized for sample handling. The holder immobilizes a crystal suspension or droplet emulsion on a nylon mesh, trapping and sealing a near-monolayer of crystals in its mother liquor between two thin Mylar films. Data can be rapidly collected in scan mode and analyzed in near real-time using piezoelectric linear stages assembled in an *XYZ* arrangement, controlled with a graphical user interface and analyzed using a high-performance computing pipeline. Here, the system was applied to two β-lactamases: a class D serine β-lactamase from *Chitinophaga pinensis* DSM 2588 and L1 metallo-β-lactamase from *Stenotrophomonas maltophilia* K279a.

## Abstract 2

A fixed-target 3D-printed mesh-holder optimized for sample handling for serial synchrotron crystallography was implemented. This allows rapid data collection from microcrystals. Data are analyzed and structures determined using a high-performance computing pipeline.

## 1. Introduction

Synchrotron serial crystallography (SSX) has emerged as a valuable approach for low-dose room-temperature structural biology research that also allows for the study of dynamic processes in protein crystals, such as chemical transformations. SSX followed and refined serial crystallography experiments (Chapman, 2019 ▸) performed at X-ray free-electron lasers (XFELs). The need arose because the powerful pulses from XFELs destroy each crystal after a single exposure, and thousands of crystals must be exposed to capture enough diffraction reflections to solve and refine the structure (Coe & Fromme, 2016 ▸; Schlichting, 2015 ▸). Improvements in technology since the introduction of serial crystallography have made SSX much more accessible and routine (Wiedorn *et al.*, 2018 ▸; Martiel *et al.*, 2019 ▸; Mehrabi *et al.*, 2020 ▸; Pearson & Mehrabi, 2020 ▸; Weinert *et al.*, 2019 ▸; Wilamowski *et al.*, 2021 ▸). SSX has become available at many microfocus synchrotron beamlines and is being widely adopted by the structural biology community.

Although the serial crystallography field has matured significantly (Chapman, 2019 ▸), challenges remain (Mishin *et al.*, 2019 ▸; Orville, 2020 ▸). Sample preparation and delivery are the critical elements in these experiments, and various approaches have been developed (Mehrabi *et al.*, 2020 ▸; Doak *et al.*, 2018 ▸; Hunter *et al.*, 2011 ▸; Lee *et al.*, 2019 ▸; Lyubimov *et al.*, 2015 ▸; Oberthuer *et al.*, 2017 ▸; Roedig *et al.*, 2015 ▸). Existing solutions for serial crystallography include: (i) liquid injectors (fast and slow) (Oberthuer *et al.*, 2017 ▸; Echelmeier *et al.*, 2019 ▸; Schulz *et al.*, 2019 ▸; Fromme *et al.*, 2015 ▸), (ii) fixed-target including silicon-nitride (Mehrabi *et al.*, 2020 ▸) and micro-patterned chips (Roedig *et al.*, 2015 ▸), (iii) microfluidic traps (Lyubimov *et al.*, 2015 ▸), (iv) nylon mesh (Lee *et al.*, 2019 ▸), (v) ‘chipless chips’ (Doak *et al.*, 2018 ▸), and (vi) tape-drives (Beyerlein *et al.*, 2017 ▸). Each method has unique advantages, such as mixing and releasing compounds, delivery in a vacuum, temperature ranges, humidity-control, and delivery speed. Drawbacks can include high sample consumption, difficulty in loading and timing considerations, as well as overall complexity that may create a barrier to use.

In addition to delivery methods and environmental options, an essential driver for serial crystallography experiments is biology and biochemistry. SSX can capture structural changes, including binding of metals, molecules and cofactors, and record chemical transformation, including enzymatic catalysis, in a time-dependent manner (Joachimiak *et al.*, 2022 ▸). Structures determined at different time points can help visualize structural and chemical changes with time scales spanning sixteen orders of magnitude, from femtoseconds (Coe & Fromme, 2016 ▸) to minutes (Weinert *et al.*, 2017 ▸). Though it still takes thousands of crystals to measure each time point, the advancements in modern light sources, delivery methods, detectors and analysis pipelines have converged to make atomic resolution structural dynamics an exciting new investigative option.

**Figure 1:**
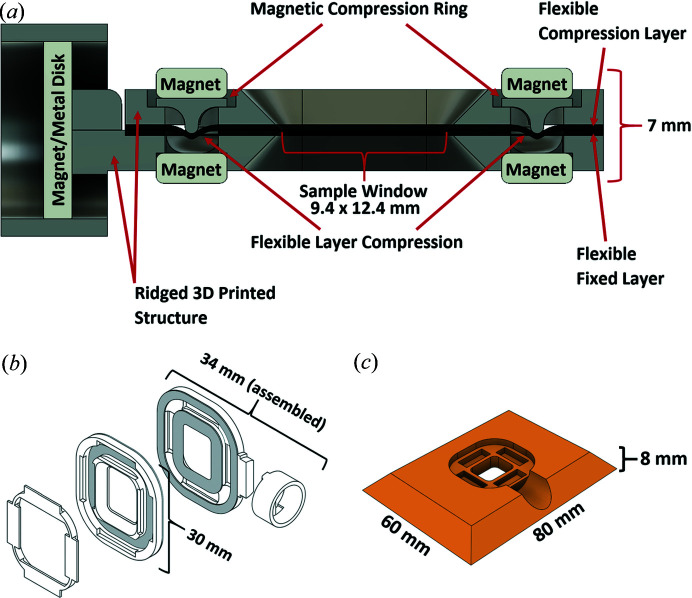
ALEX mesh-holder (larger size). (*a*) Cut-away view of the mesh-holder, (*b*) expanded isometric view (without magnets) and (*c*) isometric view of the mesh-holder loading plate.

We have recently demonstrated the benefit of the SSX approach in studies of the SARS-CoV-2 Nsp10/16 2′-O RNA methyl­transferase complex. The crystal structures of Nsp10/16 with substrates (Cap-0 analog and s-adenosyl me­thio­nine) and products (Cap-1 analog and s-adenosyl-l-homocysteine) revealed the states before and after methyl­ation, occurring within the crystals during the experiments (Wilamowski *et al.*, 2021 ▸). Our previous publication focused on a scientific case; here, we describe the approaches we have developed to better utilize light sources, X-ray beams and biological samples to meet increasing demand for reliable and user-friendly access to SSX capabilities. We report a fixed-target mesh delivery method that is simple, low-cost, accepts crystals of various sizes, and can be applied to both static and time-resolved studies. We have implemented this SSX data collection system at the Structural Biology Center (SBC) 19-ID beamline at the Advanced Photon Source (APS). Our method employs the high-precision, high-speed motorized stages introduced by Owen *et al.* (2017 ▸). This report emphasizes the newly designed holders which are 3D-printed, low-cost, reliable, and can be quickly loaded and reused. The sample environment is a Mylar–mesh–Mylar sandwich (Fig. 1 ▸), sized to enable collection of a complete diffraction dataset from a single holder under standard conditions. Crystal loading requires only a steady hand and a regular pipette; the holders are attached onto the endstation goniostat using a magnetic mount in the same fashion as a standard single-crystal crystallography pin. Data collection is controlled by simple software developed at the SBC and downstream image processing engages a supercomputer-based pipeline that carries out all necessary steps in the structure determination protocol (Vescovi *et al.*, 2022 ▸) – from image integration to phasing via molecular replacement – as fast as data are collected at the beamline.

We undertook the SSX approach having the general user community in mind such that serial and dynamic X-ray crystallography can become more accessible to the broader population of researchers. We demonstrate the successful use of our technique by highlighting crystal structures of two model proteins solved previously under cryo-conditions at the SBC – class D β-lactamase (DBL) from *Chitinophaga pinensis* DSM 2588 and L1 metallo-β-lactamase (MBL) from *Stenotrophomonas maltophilia* K279a. Since 2019, several successful SSX experiments have been performed at the SBC, including research on COVID-19 projects (Wilamowski *et al.*, 2021 ▸; Joachimiak *et al.*, 2022 ▸).

## 2. Methods

### 2.1. Beamline setup

**Figure 2:**
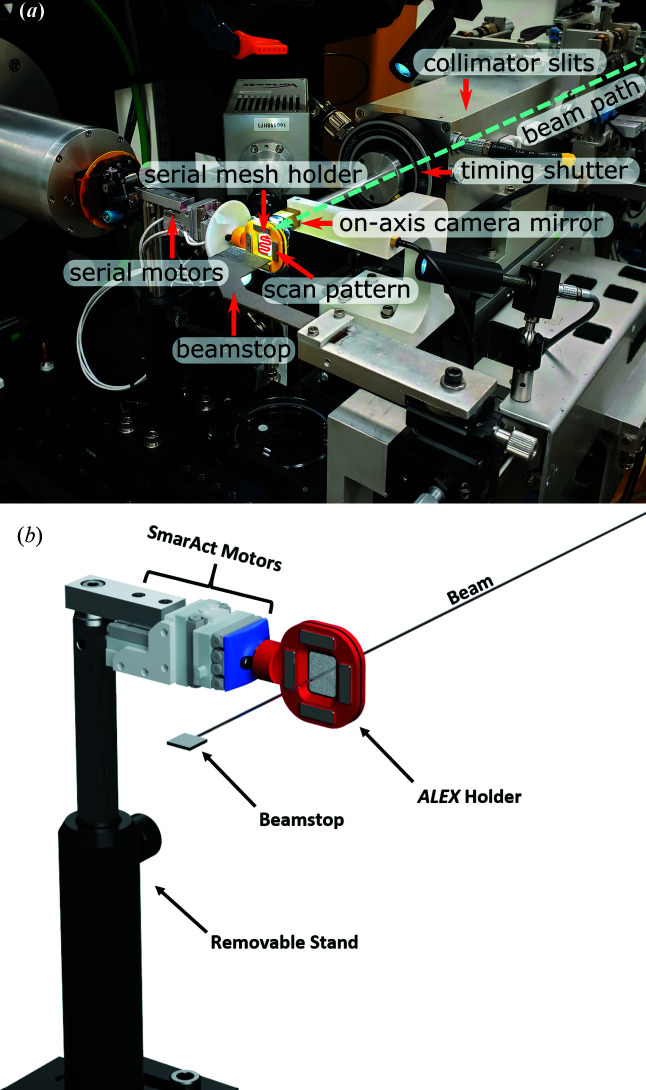
Setup of ALEX at the 19-ID beamline (*a*) using a modular magnetic mount prior to permanent integration onto the goniostat (used in data collection reported here), and (*b*) schematic of permanent integration onto the goniostat with upgraded configuration.

19-ID uses the APS undulator A to generate X-rays and a Rosenbaum double-crystal monochromator design (Rosenbaum *et al.*, 2006 ▸) with a constant X-ray beam height and a sagittally bent second crystal for horizontal focusing. The beamline is typically operated at energies between 6.0 keV and 19.5 keV. Together with the 107 mm vertical-focusing mirror, 19-ID delivers a maximum beam of 3 × 10^12^ photons s^−1^ fully focused at 40 µm (V) × 83 µm (H) which is slit down further depending on the sample requirements (Rosenbaum *et al.*, 2006 ▸). Since the collection of the data mentioned here and to minimize the impact of SSX experiments on regular beamline operations, the developed SSX motors for fixed-target stages have been permanently integrated into the endstation, rather than moved into place as needed, to be used for crystal alignment with the X-rays during ‘normal’ single-crystal experiments (Fig. 2 ▸). This setup allows collection of thousands of diffraction still images in a semi-automated scan mode. An additional upgrade since the above-mentioned experiments has been the implementation of a compact refractive lens (CRL) system, enabling beam sizing down to approximately 15 µm × 5 µm and increasing X-ray flux density by factor of ∼8 (Shu *et al.*, 2018 ▸).

### 2.2. Endstation configuration

The SBC uses SmarAct SLC-1720 piezoelectric linear stages assembled in an *XYZ* arrangement for both serial crystallography and standard cryo-crystallography sample manipulation. The *XYZ* configuration allows a maximum of 9 mm and 12 mm of travel for the *X* and *Y* directions, perpendicular to the beam, and 12 mm for *Z*, the X-ray beam direction. The system has linear range to perform various SSX experiments and is compact enough for single-crystal cryo-crystallography sample translation. The system movement is controlled via SmarAct’s SDC2 controller, which receives stepper motor signals from a Delta-Tau Turbo PMAC2 PCI ACC-24E2S stepper motor card. The detector is set to the external trigger mode, it receives an initial shutter signal from the programmable multi-axis controller (PMAC) and the data collection is carried out in a shutterless mode. X-ray shutter and detector image triggers are controlled using 5 V transistor–transistor logic (TTL) pulses from the PMAC accessory 48 I/O card. The detector can take a single or multiple images at each stopped location if a multiple serial structures (MSS) (Ebrahim *et al.*, 2019*a* ▸) or radiation damage experiment (Ebrahim *et al.*, 2019*b* ▸) is desired. A long-range cryostream retractor is integrated to vacate the sample area leaving a large open volume for SSX and other experimental work.

### 2.3. SSX sample holder and loading plate

The SBC adopted a fixed-target approach, initially with a silicon chip (Owen *et al.*, 2017 ▸) and later with a nylon mesh (this work). In the latter approach, the crystal suspension is applied on a nylon mesh and sealed between two 6 µm Mylar films. A wide range of crystal sizes can be trapped and immobilized as a near-monolayer in its mother liquor between the films. The Mylar–mesh–Mylar sandwich is held in place and sealed by a newly designed sample holder coined the Advanced Lightweight Encapsulator for Crystallography (ALEX) [Figs. 1 ▸(*a*) and 1(*b*)].

ALEX utilizes a specialized magnetic compression seal (USPO Patent Application No. 16/903,601) and is made using a dual-nozzle 3D printer. The ALEX holder is fabricated by 3D printing a flexible NinjaFlex (TPU 85A) layer followed by a ridged material (nylon, TPU-75D, *etc*.) printed directly on top. The compression ring, pin base and loading plate components are printed from only the ridged material in a separate batch. The magnets and individual pieces are then assembled manually using ep­oxy, superglue, *etc*. The sample is then loaded onto the mesh sandwiched by two sheets of Mylar using the method described below. When the two assembled sides are brought together, the compression ring is placed and secures the holder together using magnetic force while flexing the NinjaFlex layer to create an air-tight seal that runs the circumference of the sample area (Fig. 1 ▸ and Fig. S1 of the supporting information). The NinjaFlex material also provides a mildly adhesive surface which helps the Mylar remain stationary. All parts of a single holder can be 3D-printed and assembled in under an hour (Fig. 1 ▸).

A user-friendly loading plate was developed [Fig. 1 ▸(*c*)] for the ALEX holder to streamline the loading process and lower the likelihood of error and loss of sample. The impression in the loading plate is the same size as the holder with 0.5 mm of margin to ensure it can be easily placed and removed. To load the holder, the main section of the ALEX holder is placed into the impression in the loading plate, making it flush with the surface. The initial layer of Mylar is then placed with the nylon mesh following. The crystal suspension is then applied to the mesh by pipette and excess liquid is removed by blotting. The second Mylar film is laid over the mesh/solution and the second half of the holder is placed on top, resulting in the mesh and sample sealed with Mylar sheets by the properties of the holder. The magnetic compression ring is then placed, sealing and holding the entire device together, ready for data collection. Excess Mylar film at the edges can be trimmed as necessary.

Currently, ALEX holders come with two window sizes (12.4 mm × 9.4 mm or 7 mm × 7 mm) and can collect a sufficient number of diffraction images for a protein structure under normal conditions. The remaining parameters (34 mm × 30 mm × 7 mm for large or 30.5 mm × 24.6 mm × 7 mm for small, fully assembled) of the holder are physically minimized while still allowing for reliable function of the device. The center of the window is located approximately 18.5 mm (large) or 17 mm (small) from the *XYZ* stage mounting surface, which corresponds to the standard length of a crystallographic pin. Such characteristics, together with the implemented mounting base, allow for placement of the device on the same magnet as a standard pin, enabling installation as-is on other synchrotron beamlines. Sample area, distance from the base and overall geometry of the holder can be modified to allow for use on stages with longer ranges or attachments with other devices to suit various needs.

The complete setup was tested and commissioned at the 19-ID beamline with various crystal sizes (10–120 µm) and morphologies (rods, needles, cubes *etc.*; Fig. S1 of the supporting information).

## 3. Sample, purification and batch crystallization

### 3.1. Protein purification

The L1 MBL and DBL have been cloned to pMCSG7 that carries the N-terminal, removal His_6_-tag, which upon cleavage leaves three artifact residues (Ser–Asn–Ala) at the N-termini.

The *E. coli* BL21(DE3)-Gold strain (Stratagene) over expressing L1 MBL (residue 20–290) from *S. maltophilia* K279a was grown as described previously (Kim *et al.*, 2020 ▸). Specifically, a 1 l culture of enriched LB medium was grown at 37°C (190 rpm). Bacterial cells were harvested by centrifugation at 7000*g* (Sorval evolution RC centrifuge, Thermo Scientific) and frozen. Frozen cells were thawed, resuspended in a 35 ml lysis buffer [500 m*M* NaCl, 5%(*v*/*v*) glycerol, 50 m*M* HEPES pH 8.0, 20 m*M* imidazole and 10 m*M* β-mercapto­ethanol] with a protease inhibitor cocktail tablet (Complete Ultra-EDTA-free, Sigma) per litre culture, treated with lysozyme (1 mg ml^−1^) and sonicated (5 min total time, 130 W power output). Cells were spun at 30000*g* at 4°C for 1 h.

The *E. coli* BL21(DE3)-Gold strain (Stratagene) overexpressing DBL from *C. pinensis* DSM 2588 (residues 21–267, accession No. ACU58405.1) were grown at 37°C and shaken at 200 rpm in enriched M9 medium until an OD at 600 nm of 1.0 was reached, as described before (Donnelly *et al.*, 2006 ▸; Kim *et al.*, 2011 ▸). Me­thio­nine biosynthetic inhibitory amino acids (25 mg l^−1^ each of l-valine, l-isoleucine, l-leucine, l-lysine, l-threonine, l-phenyl­alanine) and l-seleno­methio­nine (SeMet, Medicillin) were added and the cultures were cooled to 4°C for 1 h before heating them to 18°C. The protein expression was induced with 0.5 m*M* iso­propyl β-d-1-thiogalactopyran­oside (IPTG). The cells were incubated overnight, harvested and resuspended in lysis buffer [500 m*M* NaCl, 5%(*v*/*v*) glycerol, 50 m*M* HEPES–NaOH pH 8.0, 20 m*M* imidazole, 10 m*M* 2­mercapto­ethanol].

Both proteins were purified by immobilized metal-affinity chromatography (IMAC-I) on an ÄKTAxpress system (cytiva, Marlborough, USA). The column was washed with 20 m*M* imidazole (lysis buffer) and eluted in the same buffer containing 250 m*M* imidazole. Immediately after purification, the His-tag was cleaved at 4°C for 20 h using a recombinant His-tagged Tobacco Etch Virus (TEV) protease. A second IMAC step (IMAC-II) was applied to remove the protease, the uncut protein and the released affinity tag.

The purified proteins were then dialyzed against a 15 m*M* HEPES pH 7.0, 100 m*M* NaCl and 2 m*M* TCEP buffer. The protein concentration was measured by UV absorption spectroscopy (280 nm) using a NanoDrop 1000 spectrophotometer (Thermo Scientific, Waltham, MA, USA). The purified proteins were concentrated using Amicon Ultra filters (Millipore, Bedford, MA, USA) to 48 mg ml^−1^ (L1 MBL) and 50 mg ml^−1^ (DBL). Individual aliquots of purified proteins were flash-cooled in liquid nitro­gen and stored at −80°C until use.

### 3.2. L1 MBL batch crystallization

We optimized batch crystallization under conditions established previously (Kim *et al.*, 2020 ▸). The L1 protein used for batch crystallization was exchanged to a buffer containing 15 m*M* Tris, 0.1 *M* KCl, 1.5 m*M* TCEP, 5 m*M* ZnCl_2_ pH 7.0 and 200 µl of L1 at 48 mg ml^−1^ was added to 200 µl of crystallization buffer: 0.15 *M* sodium malonate pH 8.0, 20%(*w*/*v*) PEG3350. Crystallizations were set up in 500 µl polypropyl­ene tubes, which were stored in horizontal position at 289 K. The crystals were harvested for SSX after approximately two weeks from setting up crystallization. One hour before SSX data collection crystals were centrifuged at 100 rpm at room temperature, excess buffer was removed until 30 µl were left in a tube. The crystals were resuspended in the remaining volume using a pipette tip with a cut end to prevent mechanical damage to the crystals. Finally, 15 µl of crystal suspension was pipetted evenly on a nylon mesh that was docked in a partially assembled ALEX chip. Subsequently, the crystals were covered with a piece of Mylar, and the chip was sealed by the top part of ALEX holder.

### 3.3. DBL crystallization in microfluidic droplets

The DBL protein was crystallized in aqueous droplets in fluorinated oil using a microfluidic setup. The microfluidic droplet generator chips were fabricated via soft lithography at the Center for Nanomaterials (Argonne, IL, USA) (Babnigg *et al.*, 2022 ▸). Small aliquots (10 µl) of the 50 mg ml^−1^ protein stock solution (in 20 m*M* HEPES–NaOH pH 8.0, 250 m*M* NaCl, 2 m*M* DTT) and crystallization screen [0.1 *M* Bis–Tris pH 9.0, 8%(*w*/*v*) PEG 20000] were used to generate droplets with Bio-Rad droplet generation oil for probes. The droplets were incubated at 16°C. Crystals of 10–100 µm were observed in droplets (50–100 µm diameter) in as little as 2 h after droplet generation. The protein concentration was optimized for crystallization in droplets (50 mg ml^−1^) and was higher than that used in the plates (40 mg ml^−1^). The droplet slurry was kept at 16°C for 4 days until data collection when a 30 µl aliquot of crystals suspension was spread onto the ALEX holder as described above for the L1 protein [Fig. S1(*a*)].

### 3.4. Data collection

#### 3.4.1. Serial synchrotron crystallography data collection

For the data collection from crystals of L1 MBL and DBL, we used the ALEX holder with a 12.4 mm × 9.4 mm window size. The X-ray beam was unattenuated and collimated to 50 µm × 50 µm with a 50 µm motor step size and an exposure area of 175 × 220 steps in *X* and *Y*, respectively. The crystal-to-detector distance was 350 mm. The total number of images from the scan was 38500 for L1 MBL, with the Pilatus3 X 6M detector collecting images with 50 ms exposure time at each scanning point. The total number of images can vary slightly between samples due to stoppages and different starting points. For the DBL from *C. pinensis*, data from only one ALEX chip was required to obtain a structure. For this protein we chose a 75 µm × 75 µm beam, 50 µm overlapping step, 40 ms exposure time and a 175 × 208 grid resulting in 36400 images.

Data collection was performed using an easy-to-use MEDM/Python graphical user interface (GUI) developed at the SBC called *Cris.py*. The software takes user values and translates them to the motion control program inside the PMAC motor controller. *Cris.py* creates two JSON files containing beamline metadata and the user-supplied information about the sample. Therefore, *Cris.py* generates all information needed for downstream analysis such as unit-cell dimensions, crystal symmetry, protein name, PDB accession code structure used for molecular replacement, experimental intent and principal investigator. Data collection statistics are summarized in Table 1 ▸ and the detailed SSX protocol is included in the supporting information.

#### 3.4.2. *Kanzus* data analysis pipeline

**Figure 3:**
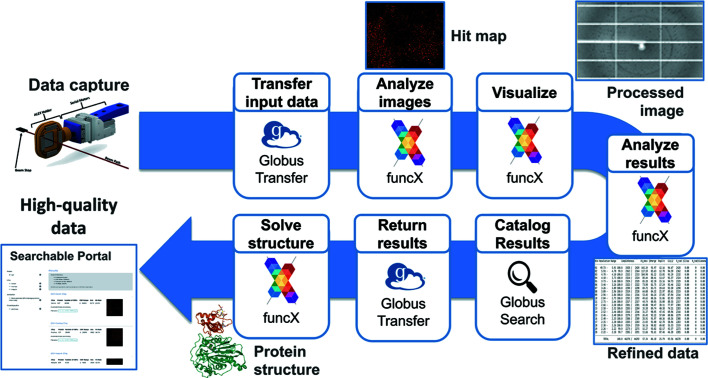
Data collection and processing pipeline. Data captured at 19ID trigger a Globus Flow to transfer images to Argonne Leadership Computing Facility where they are analyzed using the Theta supercomputer (Vescovi *et al.*, 2022 ▸). Results are visualized and published to a searchable data portal for review.

We developed a data analysis pipeline, *Kanzus*, to bridge the beamline with high-performance computing (HPC) and storage capabilities provided by Argonne Leadership Computing Facility (ALCF) and is described in detail elsewhere (Vescovi *et al.*, 2022 ▸) (Fig. 3 ▸). This pipeline manages the transfer of data, orchestration of analysis and visualization tools, and the publication of results to a user-friendly data portal. Prior to the experiment, we initiated the APS Data Management (DM) (Veseli *et al.*, 2018 ▸) system to monitor the experimental data directory and replicate data from the local directory of the beamline to a Globus accessible storage resource. This storage is connected to the ALCF by two 100 Gigabit fiber connections to accommodate rapid transfer of data between the facilities. At the ALCF we employ the Theta supercomputer to accelerate analysis and provide rapid feedback and results during the experiment. Theta is an 11.7 petaflop machine based on Intel Xeon Phi ‘Knights Landing’ (KNL), 64-core processors. Each of the 4392 nodes contains 16 GB MCDRAM and 192 GB of DDR4 RAM and leverages a high-speed InfiniBand interconnect.

We use the DM system to reliably monitor local storage, replicate data and trigger the *Kanzus* pipeline. As DM detects the creation of new files it automatically copies them to an APS-hosted storage device, where they are made available through Globus (Chard *et al.*, 2014 ▸). The DM system is configured to initiate the *Kanzus* pipeline to act on batches of 256 images. The *Kanzus* pipeline is implemented as a Globus Flow (Ananthakrishnan *et al.*, 2018 ▸). Globus Flows is a platform-as-a-service to create, share, and run distributed data management and analysis pipelines. The *Kanzus* pipeline leverages the Globus Transfer (Chard *et al.*, 2014 ▸) service to move data and the *funcX* (Chard *et al.*, 2020 ▸) function as a service platform to reliably and securely perform remote execution. *funcX* builds on the *Parsl* (Babuji *et al.*, 2019 ▸) parallel scripting library to interface with heterogeneous computing resources, allowing *funcX* to acquire compute nodes on the Theta supercomputer. We have implemented *funcX* functions for the *DIALS* (Winter *et al.*, 2022 ▸) and *PRIME* (Uervirojnangkoorn *et al.*, 2015 ▸) suites of crystallography tools with custom visualization Python scripts *Rejectoplot* and *Primalysis* to enable their use within the *Kanzus* pipeline.

In the case of the DBL datasets, the *Kanzus* pipeline performed stills processing to identify crystal lattices and integrate diffraction intensity, visualized intermediate hit-rates and metrics during chip processing, and submitted results to the beamline data portal. Two metadata JSON files created for each collection run are placed in the directory prior to data landing there and are therefore part of the first 256 files to be transferred. These metadata files are used as input to the analysis and are ingested as metadata to the portal. Since these two datasets were collected, we have extended the *Kanzus* pipeline to incorporate beam *XY* search capabilities to automatically search for optimal *XY* coordinates for the incident beam to use during analysis, and to refine the structure using the *PRIME* tool (Uervirojnangkoorn *et al.*, 2015 ▸). Later, we aim to incorporate pre-processing and triaging steps to remove unnecessary data and accelerate the time to solution.

#### 3.4.3. Structure refinement

Integrated (*int*) files from the *DIALS stills_process* were returned to the local computers for merging and scaling steps prior to the *Kanzus* pipeline upgrade a few months later. The data reported here were merged and scaled by consecutive rounds of *PRIME* and two new pieces of software, *Rejectoplot* and *Primalysis*. *PRIME* is a bulk refinement that scales all the integrated and indexed reflections. *Rejectoplot* and *Primalysis* were developed to interrogate the outputs of *PRIME*, skimming outliers (*Rejectoplot*) and making estimations for resolution cut-off (*Rejectoplot*). These two scripts are integrated into the *Kanzus* pipeline as an autonomous decision point. All processed SSX data in intensities (*I*) were converted to structure factor amplitudes (*F*) in the *Ctruncate* program (French & Wilson, 1978 ▸; Padilla & Yeates, 2003 ▸) from the *CCP4* package (Winn *et al.*, 2011 ▸) before being used for structure determination.

Both structures were solved by molecular replacement using *Molrep* (Vagin & Teplyakov, 2010 ▸) with one subunit of the reported L1 structure (PDB entry 6ua1; Kim *et al.*, to be published) for L1 and the previously refined structure of the same protein for the Class D β-lactamase as search models. These initial models were then refined by five cycles of rigid-body refinement followed by an additional ten cycles of restrained refinement using *Refmac* (Murshudov *et al.*, 2011 ▸). The structures were further refined iteratively until converged with reasonable stereochemistry using *Phenix* (Adams *et al.*, 2010 ▸) for computational refinement and *Coot* (Emsley & Cowtan, 2004 ▸) for manual adjustment. The progress of the refinement was monitored by inspecting *R* and *R*_free_ values, Ramachandran plots and *MOLPROBITY* (Davis *et al.*, 2004 ▸) throughout the process. The final structures of L1 (residues 24–287 with two zinc ions) and DBL (residues 23–267) were then validated with the RCSB validation service. The atomic coordinates and structure factors have been deposited in the Protein Data Bank under accession codes 7L52 and 7K3M for L1 and Class D β-lactamase, respectively. The final refinement statistics are given in Table 2 ▸.

#### 3.4.4. Updates to the SSX setup

The original design of the SSX system is undergoing continuous improvements. Since the initial implementation and proof-of-concept experiments, a number of features have been updated. As previously mentioned in Section 2.1, since the data mentioned here were collected the serial motors have been permanently integrated onto the goniometer. This reduced the transition time from single-crystal experiments to serial experiments from 30 min both ways, to less than 5 min. This reduction in transition time now makes performing multiple experiment types in the same scheduled beam time much more feasible as there is no longer a significant downtime. The serial motors are installed in a modular fashion which can be removed and reattached with high precision, allowing for adding-on of an already designed plates scanner system or other future equipment. This design also had the added benefit of improving the center-of-rotation alignment due to being lighter weight with less of a lever arm.

The second major addition to the beamline system was a CRL system which can focus the beam to approximately 15 µm × 5 µm and can be aligned and ready for use in under 30 min. This system greatly increases the flux density in the focal spot and can be used for both serial and single-crystal crystallography. With the smaller beam size and higher flux density, more images can be collected from an ALEX chip with the same quantity of sample.

## 4. Results

### 4.1. Development of the SSX capability at the SBC

SSX has become a valuable structural biology approach to study chemical transformations and dynamic processes in protein crystals. Sample limitations and crystal delivery to the X-ray beam represent important challenges for this approach to become more accessible to the broader scientific community. In response to the growing need for SSX and to address existing bottlenecks we have developed a new, fixed-target method implemented at the Structural Biology Center. Initial studies were conducted on crystals obtained in microfluidic droplets (Babnigg *et al.*, 2022 ▸). These successful experiments demonstrated that our SSX method is robust, performs well and is ready to be rolled out to general users.

The sample environment is provided by the ALEX holder, a small-area Mylar–mesh–Mylar sandwich designed to enable collection of a complete diffraction dataset from a single chip. The holder immobilizes and seals a near-monolayer of crystals on a nylon mesh. The developed method is simple and low-cost, accepts crystals of various sizes, and can be applied to both static and time-resolved SSX. The holders are attached to the endstation goniostat using a magnetic mount in the same fashion as a standard single-crystal crystallography pin, and data collection is controlled by simple software developed at the SBC. The ALEX accepts a small volume of microcrystal suspension which can be shipped in vials and loaded at the synchrotron and set up for data collection on-site by staff or remotely by the user. The ALEX allows data collection from tens of thousands of crystals while maintaining a biologically relevant temperature. In many cases data collected from a single chip are sufficient for structure determination. It provides reliable, user-friendly access to serial crystallography methods. The ALEX is flexible enough to transition quickly between different types of experiments.

### 4.2. Quality of SSX diffraction

**Figure 4:**
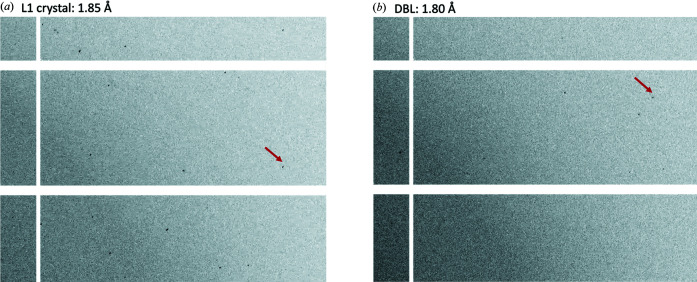
Diffraction patterns obtained from crystals of (*a*) L1 and (*b*) DBL. The arrows point to reflection at the indicated resolution.

Due to the physical geometry of the 19-ID beamline and the nature of the ALEX holder, the current method of fixed-target serial synchrotron crystallography is not optimized for low-background data collection. Much of the background scattering occurs from the beam passing through the relatively dense nylon mesh used to immobilize the crystals, of which nylon fiber diffraction can be seen in varying amounts in every diffraction image (Fig. 4 ▸). Other sources of background scattering come from the beamline itself due to its orientation and endstation components, meaning that these sources could be reduced with an improved setup and component changes.

### 4.3. Data processing and structure solution

The L1 MBL had an effective hit rate of 18.6% and 7186 indexed images were recovered. Outlier rejection was performed by analyzing the output from the post-refinement software *PRIME*, where images were rejected if they failed any of these three criteria: (i) less than 75 diffraction spots per image, (ii) resolution worse than 2.8 Å and (iii) unit-cell dimensions greater or less than 3.5% of the average [see Fig. S2 (rejectoplot)]. The resulting 6183 images were then subjected to another round of post-refinement with *PRIME* using the space group *P*6_4_22. The resolution cut-off at 1.85 Å was used following the three main metrics, I**2 rapidly increasing above 2, *CC*_1/2_ falling below 0.5 and completeness dropping to 99.9% [see Fig. S3 (primalysis)].

The DBL had a higher effective hit rate of 23.1% with a total of 8424 diffraction images prior to outlier rejection. Outlier rejection parameters were the same except for images being rejected if they had less than 25 diffraction spots. The change from 75 to 25 was due to the unit cell being significantly smaller, resulting in fewer spots per image. A total of 1163 images were rejected, resulting in 7261 moving into the final merging step. The DBL had a space group of *P*2_1_2_1_2_1_. Resolution cut-off metrics were the same as with the L1 MBL giving a final resolution of 1.80 Å.

**Figure 5:**
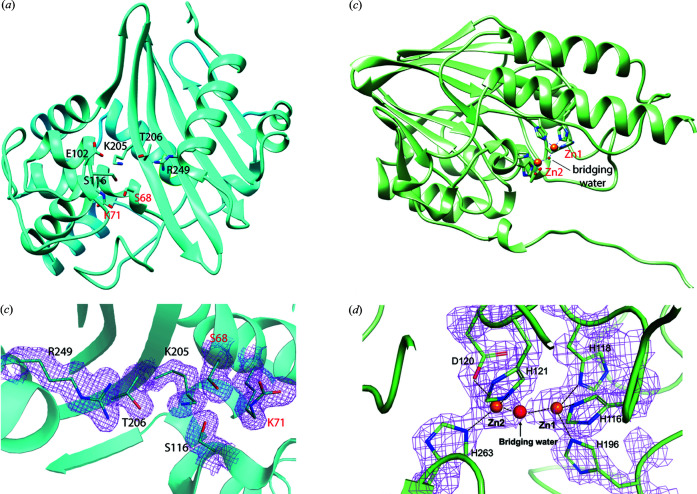
Room-temperature SSX crystal structures of class B and D β-lactamases. (*a*) Crystal structure of DBL from *C. pinensis* determined at 1.80 Å. (*b*) Crystal structure of class L1 MBL from *S. maltophilia* determined at 1.85 Å. 2*mF*_o_ − *DF*_c_ electron density maps calculated for active sites of *C. pinensis* DBL (*c*) and L1 MBL (*d*). Difference electron density maps contoured at the 1.2σ level.

The two structures were determined by molecular replacement and refined and validated as described in *Methods* [Sec sec2]. DBL from *C. pinensis* is built by a large anti-parallel β-sheet decorated by nine α-helices [Fig. 5 ▸(*a*)], while L1 from *S. maltophilia* possesses a canonical fold for MBLs with a hydro­phobic core made from two β-sheets inserted between α-helices [Fig. 5 ▸(*b*)]. Both structures are the first room-temperature models of these proteins and demonstrate great agreement with their cryogenic equivalents, including organization of their active sites. The two SSX structures superpose with their cryo counterparts with overall root mean square deviation (RMSD) of Cα atoms = 0.28 Å over 264 residues for L1 MBL and 0.29 Å over 245 residues for DBL. The structural details will be described elsewhere.

The quality of the SSX room-temperature data is illustrated by the 2*mF*_o_ − *DF*_c_ maps contoured around residues in the active site of DBL from *C. pinensis* and L1 MBL from *S. maltophilia* [Figs. 5 ▸(*c*) and 5(*d*)]. Visual inspection of the calculated 2*mF*_o_ − *DF*_c_ maps around catalytic residues shows that maps are comparable or better than cryo-crystallography structures solved at similar resolution. The system for SSX data collection is ready for structural dynamic studies of β-lactamases and other enzymes.

## 5. Conclusions

Serial crystallography has become available at XFELs and now also at light sources around the world. It provides an exciting opportunity to study enzyme structures under physiological temperatures and lower radiation dose, and investigate structural dynamics using crystallography. We responded to the increasing demand for SSX, and have developed a simple and accessible fixed-target serial crystallography approach. The key elements of this system include: (i) the reusable, 3D-printed fixed-target ALEX sample holder for the immobilization of the crystal suspension, and (ii) a robust computational pipeline for data transfer and processing utilizing the high-performance computing (HPC) resources to generate on-the-fly feedback about data quality. The applicability of the design has been demonstrated on two model proteins, which yielded the first room-temperature structures for these macromolecules with quality comparable to their cryogenic counterparts.

This SSX data collection system was implemented at the SBC 19-ID beamline at the APS. To complement the data collection, we have established the *Kanzus* software data processing pipeline linked to the HPC resources at the ALCF. The pipeline moves the data to the Globus accessible storage resource and executes analysis of diffraction images providing feedback to data collection via a user-friendly data portal.

The SSX implemented at the SBC was designed with general users in mind. The goal was to make SSX easy to access, reduce the cost of use and be simple enough as to not create a technological barrier preventing its wider adoption. The beamline systems and the ALEX holder accomplish these goals by having a specialized holder that can be 3D-printed in under an hour (protocol for 3D printing is available on request) for less than $20 in material cost, and a beamline that is flexible enough to transition quickly between different types of experiments. Our system can be applied to both static and time-resolved studies.

To provide access to general users, crystal suspension can be shipped in vials which can then be loaded on-site by SBC staff with data collection being performed by staff or remotely by the user. Other options can include users traveling on-site and performing these actions themselves with staff oversight or having fully loaded and assembled ALEX holders shipped in for remote data collection. The latter option would require creation of a shipping box which would keep the samples viable during transport. The SBC is now accepting general user proposals for beam time in a collaborative mode.

## Supplementary Material

mmcif file. DOI: 10.1107/S1600577522007895/yn5090sup1.mcf

Structure factors: contains datablock(s) r7l52sf. DOI: 10.1107/S1600577522007895/yn5090sup2.hkl

Structure factors: contains datablock(s) r7k3msf. DOI: 10.1107/S1600577522007895/yn5090sup3.hkl

Supporting figures. DOI: 10.1107/S1600577522007895/yn5090sup4.pdf

PDB reference: 7L52

PDB reference: 7K3M

## Figures and Tables

**Table 1:** Experimental setup and SSX data collection statistics

Proteins	DBL from *C. pinensis*	L1 MBL from *S. maltophilia*
Beamline	19-ID, APS	19-ID, APS
Wavelength (Å)	0.9792	0.9792
Temperature (K)	295	295
Detector	PILATUS3 X 6M	PILATUS3 X 6M
Beam size (µm)	75 × 75	50 × 50
Step size (µm)	50	50
Flux (photons s^−1^)	3 × 10^12^	3 × 10^12^
Exposure (ms)	40	50
Crystal–detector distance (mm)	350	350
Number of chips	1	1
Number of collected images	36400	38500
Number of indexed images	8424	7186
Indexing hit rate (%)	23.1	18.7
Diffraction images rejected with too low resolution	565 (2.5 Å)†	788 (2.8 Å)
Diffraction images with too-few reflections	82 (25)	218 (75)
Diffraction images greater deviation from the crystal unit-cell volume	404 (3.5%)	212 (3.5%)
Number of images after rejection of outliers	7261	6183
Images with indexed single lattice	5758	5814
Images with two indexed lattices	1293	349
Images with three indexed lattices	210	20
Lattices merged for structure determination	7018	5437
Total time of data collection (min)	80	87
Average diffraction weighted dose (kGy)	11.8	19.4

† Values in parentheses correspond to cut-off values.

**Table 2:** Data collection and structure refinement statistics

	DBL from *C. pinensis*	L1 MBL from *S. maltophilia*
PDB entry	7K3M	7L52
Space group	*P*2_1_2_1_2_1_	*P*6_4_22
*a*, *b*, *c* (Å)	49.41, 69.35, 70.04	105.67, 105.67, 99.33
Resolution range (Å)†	49.28–1.80 (1.83–1.80)	46.65–1.85 (1.88–1.85)
No. of reflections	23276 (1134)	28773 (1398)
Completeness (%)	99.99 (99.82)	99.99 (100.00)
Data redundancy	79.11 (36.99)	57.37 (29.22)
*R*_split_ (%)‡	15.19 (40.49)	19.75 (36.24)
*CC*_1/2_ §	0.973 (0.732)	0.942 (0.818)
〈*I*/σ(*I*)〉	2.81 (0.76)	4.08 (1.15)
Wilson *B* factor (Å^2^)	11.5	6.93

Structure determination		
MR initial model (PDB entry)	Complex with avibactam	6UA1

Refinement		
Resolution range (Å)	49.28–1.80 (1.88–1.80)	46.65–1.85 (1.92–1.85)
Completeness (%)	100.0 (95.0)	100.0 (100.0)
No. of reflections	22940 (2667)	28496 (2793)
*R*_work_/*R*_free_ ¶ (%)	0.178/0.224 (21.72/22.53)	17.52/21.67 (22.65/25.99)
Protein chains/atoms	2029	1998
Ligand/solvent atoms	131	179
Mean temperature factor (Å^2^)	15.62	13.42
Coordinate deviations		
R.m.s.d. bonds (Å)	0.003	0.006
R.m.s.d. angles (°)	0.580	0.761
Ramachandran plot††		
Favored (%)	96.0	96.0
Allowed (%)	4.0	4.0
Outside allowed (%)	0	0

† Values in parentheses correspond to the highest resolution shell.

‡ *R*_split_ as defined by White *et al.* (2013 ▸).

§ As defined by Karplus & Diederichs (2012 ▸).

¶ *R* = Σ*h*|*F*_o_|−|*F*_c_|/Σ*h*|*F*_o_| for all reflections, where *F*_o_ and *F*_c_ are observed and calculated structure factors, respectively. *R*_free_ is calculated analogously for the test reflections, randomly selected and excluded from the refinement.

†† As defined by *Molprobity* (Davis *et al.*, 2004 ▸).
