# Supplementary material for: Fixed-target serial crystallography at the Structural Biology Center
Source: J Synchrotron Radiat. 2022 Aug 17;29(Pt 5):1141–51. doi: 10.1107/S1600577522007895 (PMC9455217; doi:10.1107/S1600577522007895)

JOURNAL OF SYNCHROTRON RADIATION

Volume 29 (2022)

Supporting information for article:

Fixed-target serial crystallography at the Structural Biology Center

Darren A. Sherrell, Alex Lavens, Mateusz Wilamowski, Youngchang Kim, Ryan Chard, Krzysztof Lazarski, Gerold Rosenbaum, Rafael Vescovi, Jessica L. Johnson, Chase Akins, Changsoo Chang, Karolina Michalska, Gyorgy Babnigg, Ian Foster and Andrzej Joachimiak

S1. Protocol for performing fixed target SSX experiment at 19-ID

This section provides a brief overview of necessary steps required in a live experiment. While many of the specifics of each step are provided in the above sections of this paper, below is a summary of the steps in order with a brief description:

1. Perform typical beam setup including tune, guard slit scans, and beam alignment to center-of-rotation crosshairs.
    a. If CRLs are being used, beamline monochromator sagittal focus, vertical focusing mirror, downstream supports, guardslit supports, and goniometer supports must all be moved to pre-identified values (10-20 minutes)
    b. CRL lens insertion/extraction motor must then be moved into place, and monochromator tuning and guardslit tuning must be performed
    c. After a and b are completed, move beam to center-of-rotation cross hairs for use.
2. Transition beamline to Serial Mode:
    a. Disable auto-mounting robot
    b. Retract cold stream
    c. Move Gonio X prime motor as necessary
3. Ensure Data Management client is running to transfer data to supercomputing for processing
4. Load crystals into ALEX holder
5. Mount ALEX holder and align to bottom left corner
    a. In most instances, running a small test with burn paper is performed to ensure system is working properly.
6. In the *Crys.py* GUI, load all fields including crystals space group, PDB, sample name, etc.
7. Calculate and enter step size, beam size, exposure time, and detector distance into GUI
8. Press *Arm* button in GUI, which loads all necessary parameters to beamline components for collection.
9. Press *Collect* button after Arm procedure completes
10. Monitor first rows to ensure system is performing nominally
11. Repeat steps 4-10 until experiment is complete
12. After last serial sample is complete reset beamline by:
    a. Moving Gonio X prime motor to previous position
    b. Insert cold stream to previous position (0 mm)
    c. Reenable auto-mounting robot

Figure S1 Crystals deposited to nylon mesh using two alternative approaches. A. 16 µl of crystal slurry of L1 MBL from *S. maltophilia* was pipetted evenly on a nylon mesh in the partially assembled ALEX chip. Excess of liquid was blotted out and crystals were covered with layer of mylar. B. The droplet slurry containing crystals of *C. pinensis* DBL protein was obtained using the droplet generator chip [33] and the 30 µl of droplet slurry was pipetted evenly on a nylon mesh in the partially assembled ALEX chip and were covered with mylar foil. Scale bar: 50 µm.

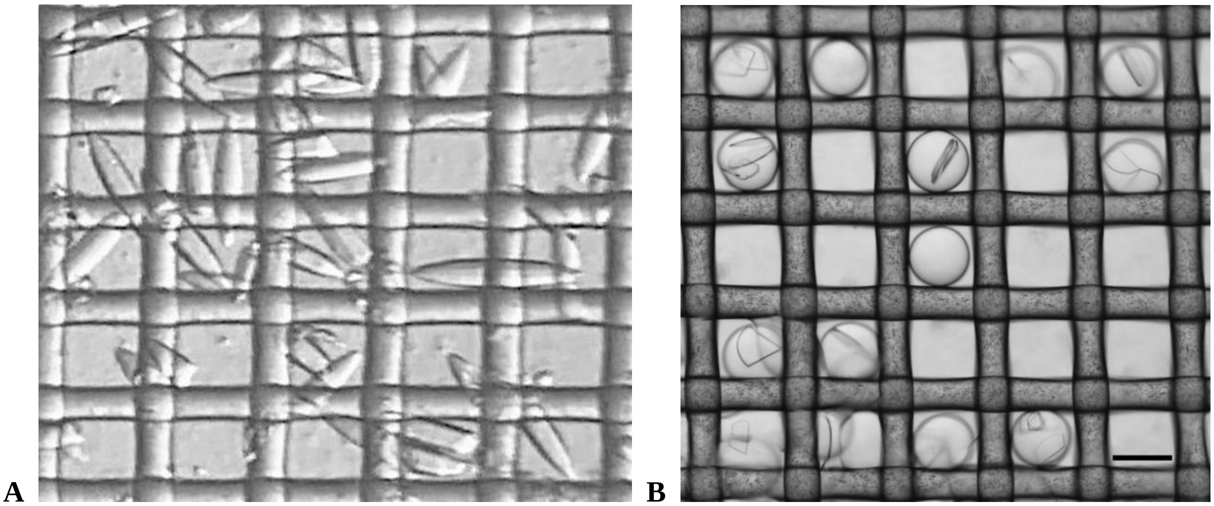

A **B**

Figure S2 Graph A. Processing of SSX data with the refinement software PRIME, histograms illustrate number of reflections, resolution and unit cell volume and variation of unit cell parameters obtained for integrated images. Depicted integration statistics is for L1 MBL. The blue bars show data before rejections of outliers (unit cell deviation > 1.6%, unit cell volume deviation > 5.4%, resolution < 2.8 Å, number of reflections < 75), orange bars depict data used for scaling and refinement of the L1 MBL structure.

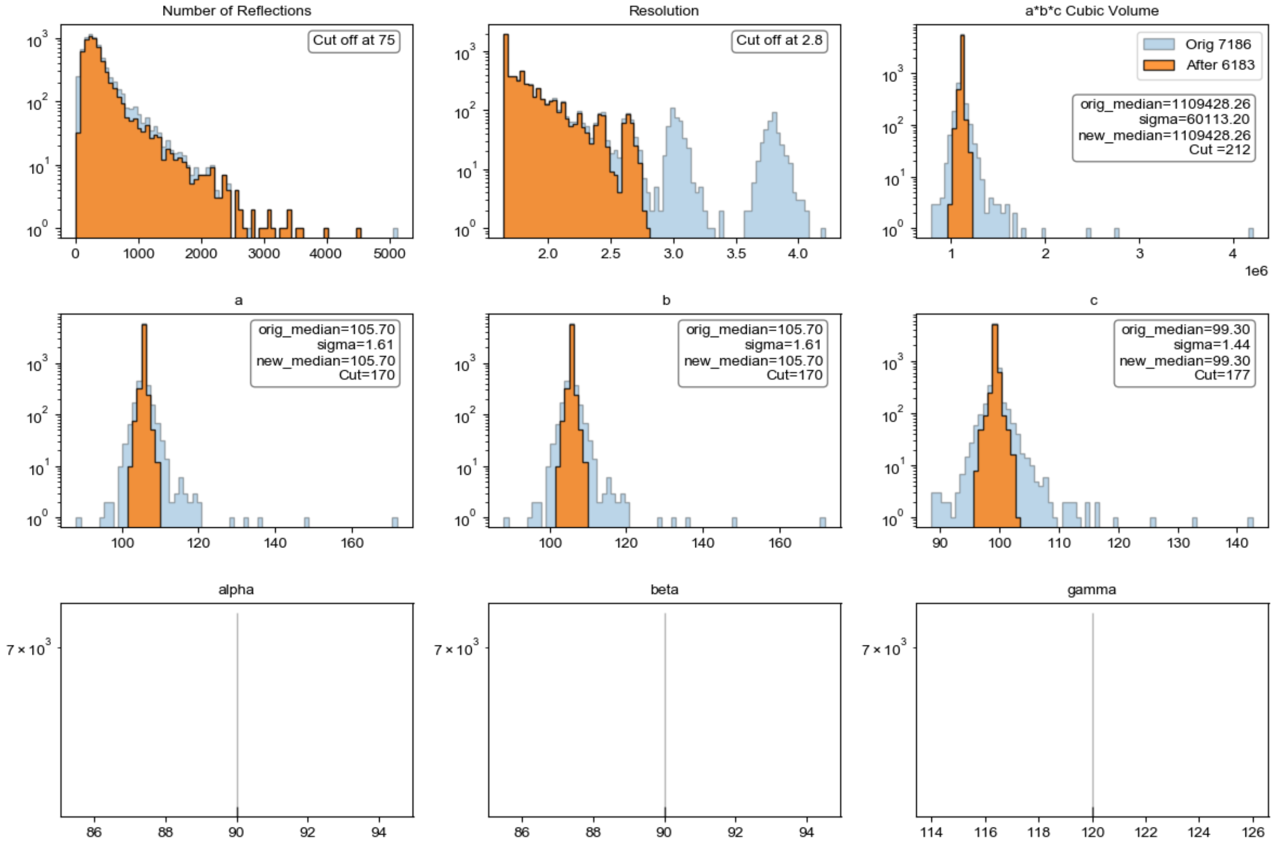

Figure S3 Graph B. The post-refinement of L1 MBL data with PRIME using space group $P6_422$, the resolution cut-off was at 1.85 Å based on the following main metrics values with resolution, I**2 rapidly increasing above 2, $CC_{1/2}$ falling below 0.5, and completeness dropping to 99.9 %.

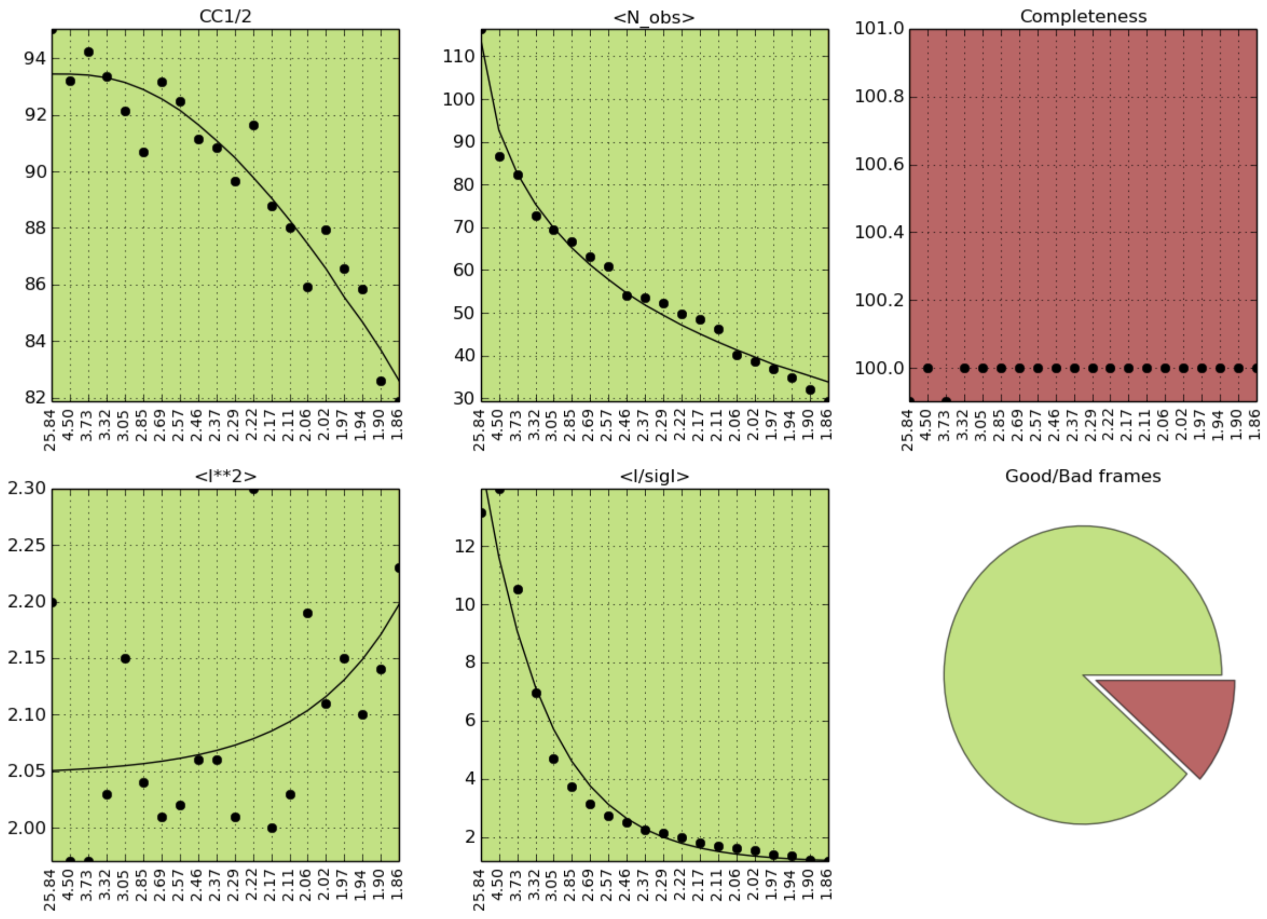

Supplement: Supplementary file 4 [file s-29-01141-sup4.pdf]
